# Projection range of eDNA analysis in marshes: a suggestion from the Siberian salamander (*Salamandrella keyserlingii*) inhabiting the Kushiro marsh, Japan

**DOI:** 10.7717/peerj.9764

**Published:** 2020-08-20

**Authors:** Daiki Takeshita, Shigeharu Terui, Kousuke Ikeda, Takashi Mitsuzuka, Maslin Osathanunkul, Toshifumi Minamoto

**Affiliations:** 1 Graduate School of Human Development and Environment, Kobe University, Kobe, Hyogo, Japan; 2 NPO PEG, Kushiro, Hokkaido, Japan; 3 Pacific Consultants Co., LTD., Chiyoda, Tokyo, Japan; 4 Department of Biology, Faculty of Science, Chiang Mai University, Amphur Muang, Chiang Mai, Thailand; 5 Research Center in Bioresources for Agriculture, Industry and Medicine, Chiang Mai University, Amphur Muang, Chiang Mai, Thailand

**Keywords:** eDNA, Marshes, Breeding, Siberian salamander (*Salamandrella keyserlingii*), Projection range

## Abstract

**Background:**

Freshwater ecosystems are rapidly declining. The Siberian salamander (*Salamandrella keyserlingii*) which inhabits the Kushiro marsh in Hokkaido, Japan has lost some habitat due to human activity. There are many challenges associated with conventional monitoring methods, including cost, the need for specialist personnel, environmental impact, and ability to detect the presence of this species; thus, we investigated the feasibility of using environmental DNA (eDNA) analysis to detect its presence and identify its breeding grounds.

**Methods:**

We performed tank experiments to confirm eDNA emission from egg sacs, larvae, and adult Siberian salamanders in the water. We also performed water sampling and visual observation of egg sacs in the Kushiro marsh during the end of the breeding season and the larval season.

**Results:**

The tank experiments found eDNA emission from all growth stages. It also implied concentrated emissions just after spawning and after hatching, and limited emissions during the incubation phase in egg sacs. We also detected eDNA in the field, likely reflecting the distribution of egg sacs or larvae. Combining this data with visual observations, it was determined that the eDNA results from the field were best explained by the number of egg sacs within 7–10 m of the sampling point.

**Conclusions:**

The results of this investigation show that the breeding sites and habitats of marshland species can successfully be monitored using eDNA analysis. They also suggest that the eDNA results from the marshes may reflect the biomass that is in close range to the sampling point. These results support the increased use of eDNA analysis in marshes and provide knowledge that could improve the interpretation of future results.

## Introduction

Freshwater ecosystems, provide habitat for at least 126,000 known species of mollusks, insects, fishes, reptiles, mammals, and plants (Balian et al., 2008), and are rapidly declining worldwide. Population abundances of mammals, birds, amphibians, reptiles, and fishes in freshwater ecosystems decreased from an average of 83% from 1970 to 2014 (Grooten & Almond, 2018). Freshwater ecosystems are strongly affected by habitat modification, fragmentation, and destruction; invasive species; overfishing; pollution; forestry practices; disease; and climate change (Grooten & Almond, 2018). In many cases, these combined threats have led to catastrophic declines in freshwater biodiversity (Collen et al., 2014; Cumberlidge et al., 2009). Most local populations of wetland species are small, isolated, and vulnerable to extinction (Møller & Rørdam, 1985; Dodd, 1990; Sjögren, 1991).

Recently, environmental DNA (eDNA) analysis has been proven to be an effective tool to monitor species. It allows for the estimation of the presence/absence (Ficetola et al., 2008), distribution (Thomsen et al., 2012), and biomass (Takahara et al., 2012) of target species via analysis of DNA found in the environment through water sampling, DNA extraction, and molecular biological methods such as PCR and next-generation sequencing. In addition, such analysis has several benefits over traditional survey methods: it has a lower cost (Evans et al., 2017), does not require morphological species identification (Ficetola et al., 2008), is environmentally non-destructive (Port et al., 2016), and can detect rare species (Goldberg et al., 2011). It has been applied to taxa such as fish (Minamoto et al., 2012), mammals, amphibians, arthropods (Thomsen et al., 2012), mollusks (Egan et al., 2013), flatworms (Hashizume et al., 2017), and plants (Scriver et al., 2015). eDNA metabarcoding can comprehensively detect eDNA of target taxa (Miya et al., 2015; Evans et al., 2016; Valentini et al., 2016). Although there is potential for eDNA analysis to be used in a wider variety of fields, to date, there have been few applications to marshes (but see Hunter et al., 2015), which are natural grasslands that develop in wet and oligotrophic areas. In addition, the range that the analysis reflects in such environment is unknown.

The Siberian salamander (*Salamandrella keyserlingii*) is one of the most widely distributed amphibian species globally (Kuzmin, 1999), but in Japan, its distribution is limited to the Kushiro marsh (Sato & Matsui, 2013) and Kamishihoro town (Matsui et al., 2019) in Hokkaido, Japan. The Kushiro marsh has become increasingly dry in recent years due to development in the surrounding land (Oki et al., 2005). The low moor consisting of reeds and sedges has been replaced by Alder forest (Oki et al., 2005). For the Siberian salamander, there have only been studies conducted with traditional survey methods, such as visual investigations of egg sacs (Hasumi & Kanda, 1998; Tazaki et al., 2008) or capture of individuals (Hasumi & Kanda, 2007).

Here, we applied eDNA analysis to Siberian salamanders inhabiting the Kushiro marsh using two qPCR assays, and (1) assessed eDNA emission from egg sacs, larvae, and adult Siberian salamander in tank experiments and (2) performed field surveys in the Kushiro marsh to estimate the projection range of eDNA analysis in a marshy environment.

## Materials and Methods

### Target species

In this study, the target species was the Siberian salamander of the family Hynobiidae. This terrestrial salamander temporarily uses still water bodies for reproduction, from mid-April to May (Hasumi & Kanda, 1998). After mating, the female lays a pair of egg sacs containing 184 ± 49.87 (SD) eggs (Terui, 2013). Larvae metamorphose between the end of July and the beginning of August, and then, the juveniles move to land (Sato, 1996). Sexual maturity takes 2–3 years for males and 3–4 years for females (Hasumi, 2010).

### Contamination prevention

To prevent contamination between samples, filtration and DNA extraction were conducted in one room and PCR in another, and a unidirectional workflow was adopted. Nitrile groves were worn during sampling and experiments. All labware was soaked in 0.1% bleach (Hospital Haiter; Kao Inc., Tokyo, Japan) for 5 min, then washed with tap water and reverse osmosis membrane-filtered (RO) water.

### Collection of water samples

We collected water samples during tank experiments and a field survey, with sterile plastic bottles. Immediately after water sampling, final volume of 0.1% (w/v) benzalkonium chloride (BAC) solution (Nihon Pharmaceutical, Tokyo, Japan) was added to each sample and the sample was mixed well to prevent eDNA degradation (Yamanaka et al., 2017). All samples were transported to the laboratory at Kobe University, Japan within 3 days of sampling.

### Tank experiments

We conducted tank experiments to assess the eDNA emission from Siberian salamander egg sacs containing eggs, larvae, and adults. Specimens were placed in tanks with aged tap water. After water sampling, egg sacs and specimens were transferred to new tanks with renewed aged tap water, whose volume was the same as the initial quantity. In each experiment, a single tank with only aged tap water was used as the negative control. The water in this tank was renewed at the same frequency as that of the other experimental treatments. All specimens were released at their corresponding collection sites after the experiment. In Japan, experiments involving amphibians do not require legal procedures or permission. However, so as not to cause pain to the specimens, experiments were carried out in accordance with the Japanese laws and guidelines governing experiments on mammals, birds, and reptiles as below; Act on Welfare and Management of Animals (Notice of the Ministry of the Environment No. 105 of October 1, 1973), Standards relating to the Care and Keeping and Reducing Pain of Laboratory Animals (Notice of the Ministry of the Environment No. 88 of 2006), Fundamental Guidelines for Proper Conduct of Animal Experiment and Related Activities in Academic Research Institutions under the jurisdiction of the Ministry of Education (Notice of Ministry of Education No. 71, 2006), and Guidelines for Proper Conduct of Animal Experiments (established by the Science Council of Japan on June 1, 2006).

Three pairs of egg sacs produced within 24 h were obtained in Kushiro town on April 22, 2019, and one pair was placed into each of three tanks (130 mm × 80 mm × 150 mm; maximum volume 1.3 L), each containing 1.0 L of water. The experiment was started on April 22. Water samples (approx. 350 mL each) were taken on April 23, April 30, May 7, and May 15.

Six male adult specimens were captured in Kushiro town on April 21 and placed into three tanks (200 mm × 200 mm × 200 mm; maximum volume: 7.0 L), each filled with 3.8 L of water, two males per tank. The experiment was started on April 22. Water samples (500 mL) were taken on April 23, April 30, May 7, and May 15. To prevent the salamanders from drowning, plastic platforms were installed in each tank on April 23. In one of the three tanks, specimens spent a lot of time outside of the water, and it was observed that sexual dimorphism had disappeared; so, water sampling was suspended before the third sampling.

Following the completion of the experiments mentioned above, fifty larvae that hatched from a single egg sac during the experiment were placed into five tanks (130 mm × 80 mm × 150 mm; maximum volume 1.3 L) containing 1.0 L of water each (ten larvae per tank). The experiment was started on May 14. Water samples (500 mL) were taken on May 22 and 29.

### Field survey

To assess the relationship between the results of eDNA analysis and a conventional method, we conducted field surveys (Fig. S1) near Ninishibetsu river in Kushiro city, Hokkaido; we avoid describing detailed geographic information for conservation reasons. Since the Siberian salamander is a protected species designated by Kushiro city, we conducted the survey with permission from the city, which manages Siberian salamander and its habitat. The whole area is low moor, mainly consisting of reeds and sedges. The average water level is about 25 cm, and the bottom sediment consists mostly of humus and mud, without gravel or sand. The area includes a Siberian salamander habitat where spawning was observed. Water sampling was conducted on May 10–11, 2017, corresponding to the end of the breeding season, when reproduction activity was expected to be low and adults were less likely to be in the water, and July 10–11, 2017, corresponding to the larval season. Grids with 10 m or 25 m intervals were designed to cover the area. The number of sampling points was 99 for May and 79 for July. At each sampling point, water was sampled following the above-mentioned procedure. The volume of sampled water was 1.0 L. Visual surveys for egg sacs were conducted on May 9–12, 2017, with six surveyors in a row exploring the area. The numbers of egg sacs were recorded.

### Filtration

Samples were filtered with two glass fiber filters (GF/F; Whatman Inc., Florham Park, NJ, USA). To evaluate contamination, RO water was filtered with two filters and treated as negative controls. The volume of RO water was 500 mL for the tank experiments, and 1.0 L for the field survey. Filters were stored at −25 °C until extraction.

### DNA extraction

Following Minamoto et al. (2019), DNA was extracted from the filters using a DNeasy Blood & Tissue Kit (Qiagen, Hilden, Germany) and Salivettes (Sarstedt, Nümbrecht, Germany) with two filters combined per sample. The extracted DNA samples, whose volume was 100 μL each, were stored at −25 °C.

### qPCR detection assays

#### Primer sets and probes

We used two qPCR assays for Siberian salamander eDNA in this study. One, which was newly designed, targeted the Cytb region, and the other targeted the 12S rRNA region (Sakai et al., 2019).

To design the new primers, we first prepared the sequence data of the Siberian salamander and the Hokkaido salamander (*Hynobius retardatus*), the only other salamander species present in the surveyed area. These data were downloaded from the National Center for Biotechnology Information (NCBI) (Table S1). Then we manually designed a species-specific primer set, “Skey_Cytb_F” and “Skey_Cytb_R” (Table 1), at the regions with the sequences that differ between these species. We conducted an in silico test with Primer-Blast (https://www.ncbi.nlm.nih.gov/tools/primer-blast/) to confirm the specificity of the primer set. Then, we created a species-specific TaqMan probe, “Skey_Cytb_P” (Table 1), using Primer Express Software v3.0 (Thermo Fisher Scientific, Waltham, MA, USA) with default settings.

**Table 1:** Primers and probes used in this study.

Name	Property	Sequence	References
Skey_Cytb_F	Forward primer	5′-GAAACTTTGGCTCACTTCTAGGAGT-3′	this study
Skey_Cytb_R	Reverse primer	5′-ACATCTCGGCAAATATGAGCCA-3′	this study
Skey_Cytb_P	Probe	5′-FAM-CAAATCGCTACAGGCCTATTTCTTGCCA-TAMRA-3′	this study
Hynobius_12S_F1	Forward primer	5′-TTAATAAAAACGGCCTAAAGCGTG-3′	Sakai et al. (2019)
Hynobius_12S_R1	Reverse primer	5′-TCAATTATAGAACAGGCTCCTCTAGGG-3′	Sakai et al. (2019)
Skey_12S_MGB_P	Probe	5′-FAM-ACTTTGGAAACCCCGCC-MGBEQ-3′	this study

For 12S, we used an existing primer set (Sakai et al., 2019). This primer set was originally designed for *Hynobius* species, but an in silico test with Primer-Blast, confirmed that there is no substitution with the sequence of the Siberian salamander. We designed a new species-specific probe, “Skey_12S_MGB_P” (Table 1), with Primer Express Software v3.0 set to default parameters.

### In vitro specificity assessment

We assessed the specificity of the detection assays using an eDNA sample taken from a tank with adult specimens (described later; Table S2) and a sample of DNA extracted from Hokkaido salamander eggs. DNA was extracted with a DNeasy Blood & Tissue Kit (Qiagen, Hilden, Germany) following the manufacturer’s instructions. The DNA concentration in the extract was measured with Qubit 3.0 (Thermo Fisher Scientific, Waltham, MA, USA). Dilute solutions (50 pg/μL and 5 pg/μL) of Hokkaido salamander DNA were made using TE buffer.

To evaluate the specificity of the Cytb detection assay, we conducted real-time PCR with StepOnePlus (Thermo Fisher Scientific, Waltham, MA, USA). The total volume of the PCR mixture was 20 μL; this contained 1 × TaqMan Environmental Master Mix (Thermo Fisher Scientific, Waltham, MA, USA), 2.0 μL of DNA samples, 0.1 μL of AmpErase Uracil N-Glycosylase (Thermo Fisher Scientific, Waltham, MA, USA), 900 nM each of Skey_Cytb_F and Skey_Cytb_R, and 125 nM of Skey_Cytb_P. PCR was conducted with DNA samples from the two salamander species and a negative control (pure water instead of DNA template) in triplicate. The thermal conditions were: 50 °C for 2 min, 95 °C for 10 min, and 55 cycles of 95 °C for 15 s and 60 °C for 1 min.

To assess the specificity of the detection assay for 12S, we conducted real-time PCR with the 12S assay (primers Hynobius_12S_F1 and Hynobius_12S_R1 (Sakai et al., 2019), and probe Skey_12S_MGB_P). Concentration and volume of the primer set and the probe were the same as above. PCR was conducted in triplicate, with DNA samples from the two salamander species and a negative control. The thermal conditions were: 50 °C for 2 min, 95 °C for 10 min, and 55 cycles of 95 °C for 15 s, 60 °C for 1 min, and 72 °C for 1 min.

For each specificity assessment, detection was considered successful when a DNA amplification curve was observed. The same detection criteria were applied for the quantitative PCR described below.

### Quantitative PCR

We conducted qPCR with the two assays. First, we made a standard for Cytb assay from plasmids including an artificial salamander Cytb gene, and diluted it to 1.5 × 10^4^, 1.5 × 10^3^, 1.5 × 10^2^, and 1.5 × 10^1^ copies/µL. We repeated the same protocol for the 12S assay.

For samples of the tank experiments, we used the Cytb assay. The standard, eDNA samples, and negative controls including a PCR control (pure water instead of extracted eDNA) were included in the qPCR, all in triplicate, with the same conditions as those of the Cytb assay in specificity assessment. When a DNA amplification curve was not observed in any of the triplicates, these samples were re-run with 5.0 μL extracted eDNA samples (Table S2).

For field samples, we used both assays. eDNA samples, the standard, and negative controls including a PCR control were included in qPCR, all in triplicate. For the Cytb assay, the PCR conditions were the same as above. To confirm whether the amplified signal of DNA was due to the eDNA of the Siberian salamander, Sanger sequencing of amplicons was performed by FASMAC Inc., Kanagawa, Japan. For the 12S assay, the PCR conditions were the same as those of the 12S assay in terms of specificity assessment, except we used 5.0-μL extracted eDNA samples instead of 2.0-μL.

The number of eDNA copies per reaction was the average of the quantitative values of three wells. Non-amplified wells were included in the calculation as 0 copy (Ellison et al., 2006). The amount of eDNA in the water sample, being equal to that of whole extracted DNA (100 μL), was obtained by multiplying the number of eDNA copies per reaction by 50 (template volume: 2.0 μL) or 20 (5.0 μL). Finally, the concentration per liter was calculated according to the volume of each water sample.

### LOD and LOQ measurements

For each assay, the limit of detection (LOD) and the limit of quantification (LOQ) were measured. The concentration of the standards was adjusted to 3,000, 300, 30, 15, 10, 5, 3, and 1 copies per reaction. For each assay, PCR was performed in triplicate, with the standard and a negative control. PCR conditions were as mentioned above. LOD and LOQ were the lowest eDNA concentrations (copies per reaction) at which DNA amplification was observed in one of the triplicates and in all of the triplicates, respectively.

### Inhibition assessment

For the field samples, PCR inhibition was assessed using each assay. As the internal positive control, 3,000 copies per reaction of target DNA were added. For each assay, the Ct value of each field sample with the standard were compared to that of the standard only. Following Hartman, Coyne & Norwood (2005), PCR inhibition was diagnosed when ΔCt ≥ 3.

### Statistical analyses

#### Comparison of real-time PCR assays

For all the samples in field surveys, patterns of detection/non-detection were compared between the assays, using the McNemar test. For samples in which eDNA was detected with both assays, the number of eDNA copies was compared using Wilcoxon signed rank test. These tests were carried out with R version 4.0.0 (R Core Team, 2020).

#### Spatial regression analysis for eDNA concentration and number of egg sacs

For the field samples collected in May, a spatial regression analysis was performed to evaluate whether eDNA concentration was explained by the number of egg sacs. The explanatory variable was the number of egg sacs within *X* (m) (*X* = 1–150) of each water sampling point. This was based on the data of the position and the number obtained during the field survey (Table S3; Fig. S2). With QGIS 3.2.1-Bonn (QGIS Development Team, 2018), circular buffers with given radii were generated from each water sampling point and the number of egg sacs in each buffer was counted. The response variable was the log10-transformed eDNA concentration (copies/L). Notably, only the points where eDNA was detected were included in the analysis. A spatial error model (SEM) was adopted to consider the spatial autocorrelation, with *Distance Based Neighbors within k km* for the element *w* of the spatial weight matrix (*k* = 0.01, 0.025, 0.05, 0.1). After that, Akaike information criterion (AIC) was calculated for all models. The regression of the SEM and the calculation of AIC were performed with R version 4.0.0 (R Core Team, 2020).

## Results

### Establishment of eDNA detection assays

There were positive amplifications of the eDNA sample of Siberian salamander, but not of the genomic DNA sample of Hokkaido salamander (100 pg/reaction). Therefore, both assays were specific to the Siberian salamander.

### Tank experiments

**Figure 1:**
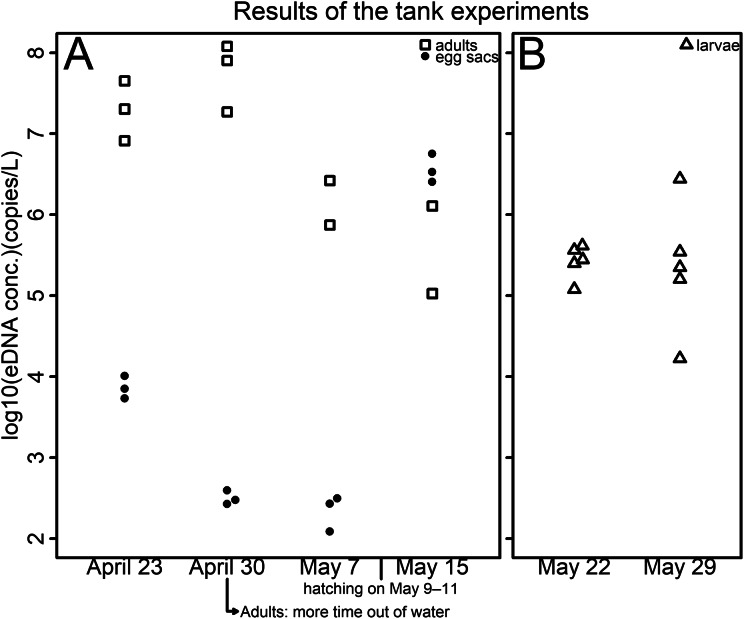
Results of the tank experiments. (A) Egg sacs and adults (B) larvae. The vertical axis indicates the log10-transformed eDNA concentration—open squares represent adults, dots represent egg sacs and open triangles represent larvae. Each tank contained either a pair of egg sacs, two male adults, or ten larvae—all life stages were housed in separate tanks. Hatching was observed on May 9, 10, and 11 in each tank. To prevent adults from drowning, plastic platforms were installed in each tank on April 23. In 1 of the 3 tanks, specimens spent a lot of time outside of the water, and sexual dimorphism had disappeared, so water sampling was suspended before the third sampling.

eDNA was detected with the Cytb assay in all samples of egg sacs, larvae, and adults (Fig. 1; Table S2). Notably, the eDNA concentration increased after the eggs hatched (Fig. 1; Table S2). The ranges of *y*-intercept, *R*^2^ values of calibration lines, and PCR efficiency were 39.749–40.958, 0.995–0.998, and 93.365–98.012%, respectively. Incidentally, eDNA was detected in some negative controls taken from the control tank (Table S2). However, the amount of eDNA detected in the negative controls was less than one-tenth of that found in other samples on the same sampling date (Table S2). Therefore, the results were accepted as they stand.

### Field survey

#### qPCR with Cytb assay

**Figure 2:**
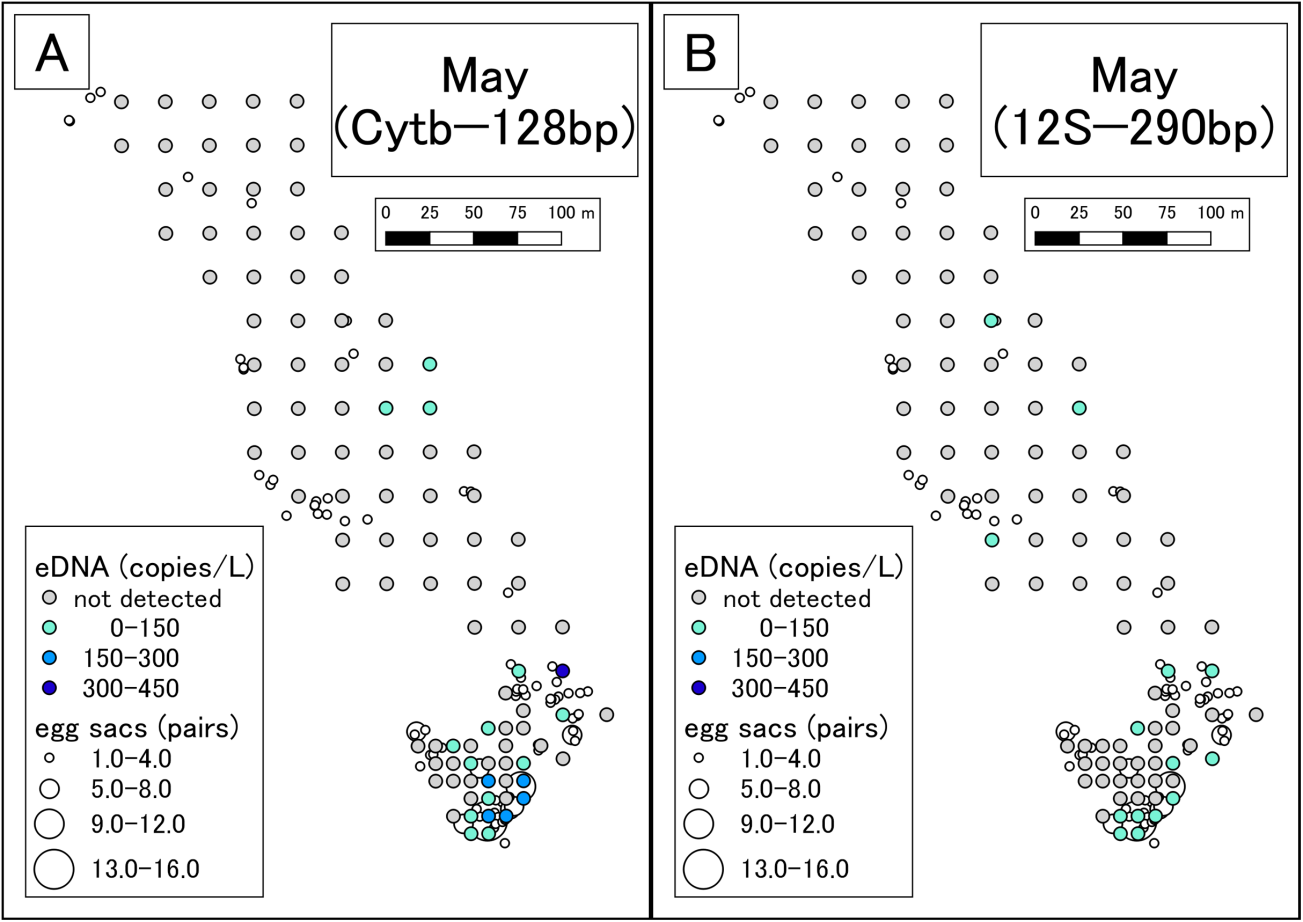
eDNA analysis and observation of egg sacs in May. (A) Results of Cytb detection assay. (B) Results of 12S detection assay. This study was conducted in the habitat of the Siberian salamander. For conservation purposes, it was necessary to conceal the specific geographical information of the survey location, such as longitude, latitude, or characteristic features. Therefore, we used a schematic map.

**Figure 3:**
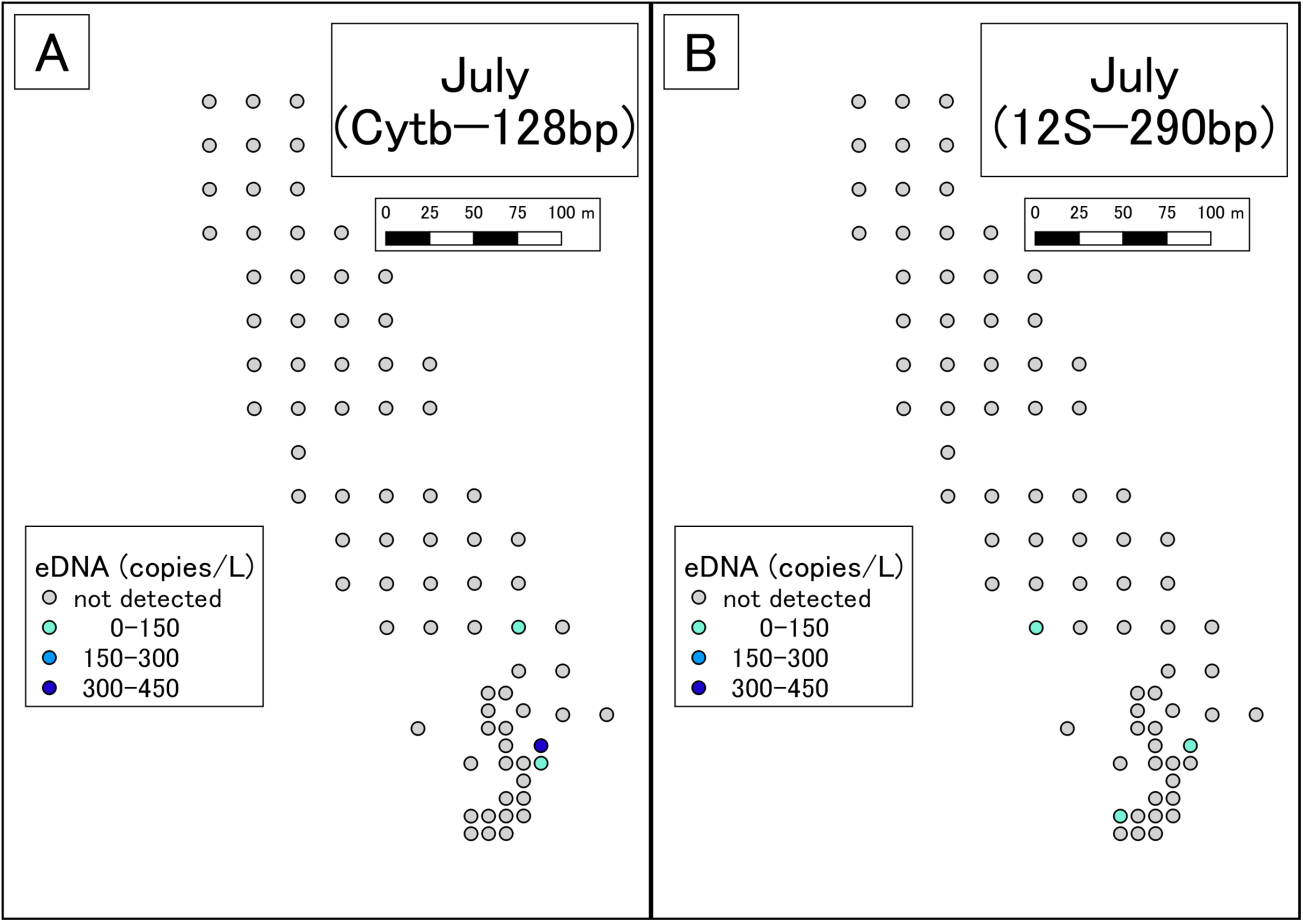
Results of eDNA analysis in July. Though some sites had dried out since May, we used as many sampling points as possible. Points plotted here show the actual sampling sites. (A) Results of Cytb detection assay. (B) Results of 12S detection assay. This study was conducted in the habitat of the Siberian salamander. For conservation reasons, it was necessary to conceal the specific geographical information of the survey location, such as longitude, latitude, or characteristic features. Therefore, we used a schematic map.

DNA amplification was observed in qPCR of 19 of the 99 May samples (Table S4; Fig. 2). The sequences were confirmed as those of the Siberian salamander in 18 samples. Sequencing failed in the one remaining sample. DNA amplification was observed in qPCR of 3 of the 79 July samples (Table S5; Fig. 3). Siberian salamander sequences were confirmed in all three samples. The ranges of *y*-intercept, *R*^2^ values of calibration lines, and the PCR efficiency were 40.740–41.723, 0.991–0.998, and 89.258–97.980%, respectively.

#### qPCR with the 12S assay

DNA amplification was observed in qPCR of 14 of the 99 May samples (Table S6; Fig. 2). DNA amplification was observed in qPCR of 3 of the 79 July samples (Table S7; Fig. 3). The ranges of *y*-intercept, *R*^2^ values of calibration lines, and the PCR efficiency were 40.747–44.397, 0.859–0.981, 89.739–106.969%, respectively.

#### Contamination issues for field samples

As above, samples of the tank experiments were contaminated. However, the field survey and the molecular experiments for field samples had been completed before the start of the tank experiments and no positive signal was observed from the negative controls of the field experiment. Therefore, the results of the field samples were not affected by the contamination.

#### LOD and LOQ measurements

For the Cytb assay, DNA amplification was observed in triplicates in as little as 5 copies, while one and two replicates were amplified in 1 and 3 copies, respectively. Thus, the LOD and LOQ were determined as 1 copy and 5 copies per reaction, respectively. However, when using the standard curve created from the results of 3,000 to 5 copies, the re-calculated mean copy number of the triplicates of 3 copies and 1 copy was 1.74 and 0.84 copies, respectively, when non-amplified replicates were treated as 0 copy (Ellison et al., 2006). These values were not far from 3 copies and 1 copy. Therefore, quantifying the eDNA amount below the LOQ should be meaningful. The *y*-intercept, the *R*^2^ value of the calibration line, and the PCR efficiency calculated from the result of 3,000 to 5 copies were 40.900, 0.993, and 100.269%, respectively.

For the 12S assay, DNA amplification was observed in triplicates in as little as 15 copies. Two replicates for 10 copies, one for 5 copies, and two for 3 copies were amplified, whereas 1 copy was not amplified. Thus, the LOD and LOQ were determined as 3 and 15 copies per reaction, respectively. The values of 10, 5, and 3 copies were recalculated in the same manner as Cytb, resulting in 10.70, 3.00, and 1.26 copies, respectively. Therefore, quantifying the eDNA amount below the LOQ should also be meaningful for 12S. The *y*-intercept, the *R*^2^ value of the calibration line, and the PCR efficiency calculated from the result of 3,000 to 15 copies were 43.604, 0.984, and 91.894%, respectively.

#### Inhibition assessment

For the Cytb assay, all field samples were characterized by ΔCt < 3, hence they were judged as non-inhibited. For the 12S assay, inhibition was partially observed. In the samples from May, 95, 3, and 1 sample(s) were defined by ΔCt < 3, ΔCt ≧ 3, and no amplification, respectively. In samples collected in July, all samples were characterized by ΔCt < 3. As a whole, there was only a small proportion of samples (4/178) with inhibition; so, the results of the eDNA analysis were used as they were in the analysis of detection/non-detection and that using the quantitative value.

### Statistical analyses

#### Comparison of detection assays

The patterns of detection/non-detection were not significantly different between the two PCR detection assays (McNemar’s chi-square test = 1.07, df = 1, *p* = 0.30). The number of eDNA copies found with the Cytb detection assay was significantly larger than that for 12S (Wilcoxon signed rank test, *Z* = 2.67, *p* = 0.005).

#### Relationship between eDNA concentration and the number of egg sacs

For both assays, eDNA concentration was significantly/marginally significantly explained by the number of egg sacs around each water sampling point. The results were similar, regardless of the value of *k* (km), considering the spatial autocorrelation, and the AIC became smaller when *X* (m) was 7–10 (Table S8). Therefore, the eDNA concentrations were best explained by the number of egg sacs within a 7–10 m radius of the sampling point. The models with the lowest AIC had *k* = 0.05 and *X* = 7 for the Cytb assay, and *k* = 0.025 and *X* = 10 for the 12S assay (Tables 2; Table S8).

**Table 2:** Minimum AIC models for each *k*-value of SEM. For sites where eDNA was detected, Spatial Error Models (SEMs) were constructed with the number of egg sacs within *X* (m) around each site as the explanatory variable and log10 (eDNA concentration) (copies/L) as the response variable. As the spatial weight matrix, *Distance Based Neighbors within k km* was adopted (*k* = 0.01, 0.025, 0.05, 0.1). Parameters of *X*, *p*-value, and AIC were described for the models with the smallest AIC at each *k*-value.

Assay	*k* (km)	*X* (m)	*p-*value	AIC
Cytb–128 bp	0.01	9	0.03	24.523
	0.025	7	0.006	23.691
	0.05	7	0.005	21.935
	0.1	7	0.003	23.045
12S–290 bp	0.01	10	0.02	44.400
	0.025	10	0.02	44.086
	0.05	10	0.06	44.584
	0.1	10	0.03	44.463

## Discussion

We succeeded in observing eDNA emission from Siberian salamander egg sacs, larvae, and adults. In addition, we detected eDNA in the field at both the end of the breeding season and the larval season, likely reflecting the distribution of egg sacs and larvae. Relating the findings of the eDNA analysis to a conventional method (visual observation), it was found that the results of the eDNA analysis were best explained by the number of egg sacs within 7–10 m of the sampling point.

### eDNA emission from each growth stage of Siberian salamanders

The eDNA concentration in tanks of egg sacs was high just after egg introduction (Table S2; Fig. 1). Because eDNA can come from feces, molted skin, organelles, or gametes (Ficetola et al., 2008; Barnes & Turner, 2016), initially, the eDNA detected may have mainly been from cells of maternal individuals or sperm adhering to the surfaces of the egg sacs. The eDNA concentrations were then found to decrease (Table S2; Fig. 1). The eDNA emissions from the embryos might be limited due to their jelly layer or the egg sac membrane. However, they were found to increase again after the eggs hatched (Table S2; Fig. 1). This may be because the eDNA that had accumulated in the egg sacs was released upon hatching. These results are in line with those of Takeuchi et al. (2019), who found that, in the Japanese eel (*Anguilla japonica*), there were no eDNA emissions observed in the fertilized egg stage, but eDNA emissions were found after hatching.

The results of the two sample dates of the larval experiment were similar (Table S2; Fig. 1). Larval eDNA emission might not be affected by the growth of the specimens. eDNA detected from adult salamanders decreased over time (Table S2; Fig. 1). Although no quantitative data was collected for out-of-water time, salamanders were observed spending more time out of water as the experiment progressed, which could explain these results. The experiments with both egg sacs and adults may indicate that the amount of eDNA emitted from adults is higher than that from egg sacs in the period when they coexist, that is, during the peak of the breeding season.

### Detection of Siberian salamander eDNA in the field

In May, more eDNA was detected in southern part of the study area than in the northern area. There was also a higher number of egg sacs in the southern area than in the north. As such, the results of eDNA analysis appeared to reflect conventional surveys. In the northern area, eDNA was detected at some sites where no egg sacs were encountered (Fig. 2). It might be that egg sacs were overlooked or that eDNA from post-spawning adult individuals was detected. However, we did not find recently laid egg sacs or adult individuals by visual observation. The breeding season in the study area was likely to have already ceased; so, any effect from post-spawning adults was thought to be minimal. We could know how the results of eDNA are affected by adults by recording adult abundance over time, including during the peak of reproduction, as performed by Buxton et al. (2017). On the contrary, there were many sites where eDNA was not detected but egg sacs were present. Since hatching was not confirmed, the egg sacs could have been in the incubation phase at the time of surveying, which implies that the peak of eDNA occurring after laying or hatching of the eggs (Fig. 1) would have been missed.

In July, Siberian salamander eDNA was detected at five sites. Two of these sites were very close together, so the five sites could be considered four clusters. Given that the study area had still waters, it appears that these clusters represent independent eDNA sources. July is the larval season for the Siberian salamander, and therefore we conclude that there were four independent clusters of larvae in the study area.

### Estimation of the projection range of eDNA analysis in marshy environments

We adopted a statistical analysis that considered spatial autocorrelation. Even though the setting of spatial autocorrelation was changed, eDNA concentration was commonly explained by the number of surrounding egg sacs (Table S8). This shows that eDNA analysis can be an effective tool in estimating the locations of Siberian salamander breeding sites. Generally, the number of egg sacs within 7–10 m of each water sampling point significantly explained the detected eDNA copy numbers (Table S8). It can be inferred that eDNA contained in a water sample taken from a marsh could be mainly affected by biomass within 7–10 m of the sampling point.

### Comparison of detection assays

The patterns of detection/non-detection were not significantly different between the Cytb and 12S assays. Both assays can be used to detect Siberian salamander eDNA for monitoring purposes. As two assays showing low LOD were used to perform the PCR, with three replicates each, it can be said that six pseudo replicates were performed. Therefore, detection/non-detection of the eDNA was thus considered to be robust in the field samples. Quantitative values of eDNA were different between the two detection assays. The Wilcoxon signed rank test results could be attributed to amplification lengths of the two detection assays: 128 bp for Cytb and 290 bp for 12S. Shorter amplification lengths can detect more degraded DNA (Deagle, Eveson & Jarman, 2006).

### Possible issues and future prospects

This study expanded the applications of eDNA analysis in marshes and demonstrated its usefulness. It also estimated the range to which the results of eDNA analysis were closely linked in the marshes. Future tank-based investigations could more closely imitate the species natal environment. This will enable improved and more accurate estimations, of the range around the sampling points that affects the results.

However, there is still room for improvement to enhance the use of eDNA analysis for species monitoring in marshes. Based on our study, one issue concerns the determination of the breeding sites and the other is about sampling optimization. Regarding the former, the present study minimized the impact of adult individuals because we chose the period when most of the adults had left the water body. However, during the peak of reproduction, it will become difficult to distinguish whether the eDNA is due to eggs or adults. Therefore, it is important to set the time of the survey appropriately for studying breeding sites. If the target species of a survey spends most of the time in the water, it will be necessary to consider focusing on the spawning-related fluctuations in eDNA emissions. Bylemans et al. (2017) showed that in perch, ratios of nu-eDNA to mt-eDNA significantly changed due to spawning. If we also find this pattern in amphibians or reptiles living in marshes, and eDNA analysis is conducted with both mitochondrial and nuclear markers, the ratio may be another useful index of breeding sites. As for sampling optimization, because eDNA was not detected at some sites with egg sacs, water sampling should be optimized in terms of spatial/temporal intervals to capture evidence of reproduction without exception.

Nonetheless, the impact of this study, which estimated the projection range of eDNA analysis in marshes for the first time, is significant. In a river, Itakura et al. (2020) indicated that the eDNA concentration of eels would reflect the abundance of the species 50 and 70 m beyond the water sampling points in lower and upper reaches of the river respectively. In the sea, the results of eDNA analysis reflected species composition within 60 m (Port et al., 2016) or fish biomass within 150 m (Yamamoto et al., 2016) of the sampling point. A marsh has little inflow and outflow of water compared to rivers, and the water column is not mixed horizontally or vertically like in the sea or large lakes. With that in mind, the scale of 7–10 m indicated by our study seems reasonable.

With the increasing number of applications of eDNA analysis, the occurrence of its use in marshes will likely increase. It will also be used not only for academic purposes, but also for environmental impact assessment or regular monitoring of endangered species by governments, as described previously by Biggs et al. (2015) or Lewis, Griffiths & Wilkinson (2016). In addition, the standardization of methods has already been promoted for ponds (Harper et al., 2019); hence, its promotion may also occur for marshes in the future.

## Conclusions

In this study, we established a new species-specific primer set and probes for Siberian salamander eDNA. We observed eDNA emission from all developmental stages (egg sacs, larvae, and adults) with concentrated emissions after spawning and after hatching, and limited emissions during the incubation phase in egg sacs in tank experiments. We estimated from the field survey that eDNA contained in a water sample taken from a marsh could be mainly affected by biomass within 7–10 m of the sampling point. We showed that eDNA analysis can be an effective tool in estimating the distribution of larvae and, more strongly, breeding grounds of the Siberian salamander. We hope that the knowledge gained in this study, especially about the projection range of eDNA analysis, will be useful in the advancement of species monitoring by eDNA analysis for marshy species, not only for the salamander, but also other animals or plants.

## Supplemental Information

10.7717/peerj.9764/supp-1 Supplemental Information 1 Study area. The red circle indicates the study area. This figure is based on the English map of Geospatial Information Authority of Japan (https://maps.gsi.go.jp/development/ichiran.html#english).

10.7717/peerj.9764/supp-2 Supplemental Information 2 Distribution of egg sacs in the study area. The size of the circle is changed according to the number of egg sacs found at each point. The identification number (1–77) corresponds to the “Site” of Supplemental Table S3, and by comparing it, the number of egg sacs and the time of discovery can be seen.

10.7717/peerj.9764/supp-3 Supplemental Information 3 Sequence data used for the new primers and probes. These data were downloaded from National Center for Biotechnology Information (NCBI).

10.7717/peerj.9764/supp-4 Supplemental Information 4 Results of the tank experiments. See the legend of Figure 1 for details.

10.7717/peerj.9764/supp-5 Supplemental Information 5 Results of observation of egg sacs. Site numbers (1–77) correspond to those of Supplemental Figure S2.

10.7717/peerj.9764/supp-6 Supplemental Information 6 Results of May field samples using the Cytb detection assay. May NC1–7 are the filtration negative controls. Sequencing failed in May95. The eDNA copy number per reaction was the average of the quantitative values of 3 wells. Non-amplified wells were included in the calculation as 0 copy (Ellison et al., 2006).

10.7717/peerj.9764/supp-7 Supplemental Information 7 Results of July field samples using the Cytb detection assay. July NC is the filtration negative control. The eDNA copy number per reaction was the average of the quantitative values of 3 wells. Non-amplified wells were included in the calculation as 0 copy (Ellison et al., 2006).

10.7717/peerj.9764/supp-8 Supplemental Information 8 Results of May field samples using the 12S detection assay. May NC1–7 are the filtration negative controls. The eDNA copy number per reaction was the average of the quantitative values of 3 wells. Non-amplified wells were included in the calculation as 0 copy (Ellison et al., 2006).

10.7717/peerj.9764/supp-9 Supplemental Information 9 Results of July field samples using the 12S detection assay. July NC is the filtration negative control. The eDNA copy number per reaction was the average of the quantitative values of 3 wells. Non-amplified wells were included in the calculation as 0 copy (Ellison et al., 2006).

10.7717/peerj.9764/supp-10 Supplemental Information 10 All Spatial Error Models (SEMs) in this study. For sites where eDNA was detected, Spatial Error Model (SEM) was constructed with explanatory variables: the number of egg sacs within X [m] around each site and response variable: log10 (eDNA concentration) (copies/L). As the spatial weight matrix, *Distance Based Neighbors within k km* was adopted. The values of *k* were 0.01, 0.025, 0.05, and 0.1.

## Additional Information and Declarations

### Field Study Permissions

The following information was supplied relating to field study approvals (i.e., approving body and any reference numbers): Field experiments were approved by the Kushiro City for water sampling and visual observation of egg sacs.

### Data Availability

The following information was supplied regarding data availability: The raw measurements are available in Tables S2–S7 which contain measurements for tank experiments, observation of egg sacs, and field samples, respectively.
