# Supplementary figures and images for: Projection range of eDNA analysis in marshes: a suggestion from the Siberian salamander (Salamandrella keyserlingii) inhabiting the Kushiro marsh, Japan
Source: PeerJ. 2020 Aug 20;8:e9764. doi: 10.7717/peerj.9764 (PMC7443320; doi:10.7717/peerj.9764)

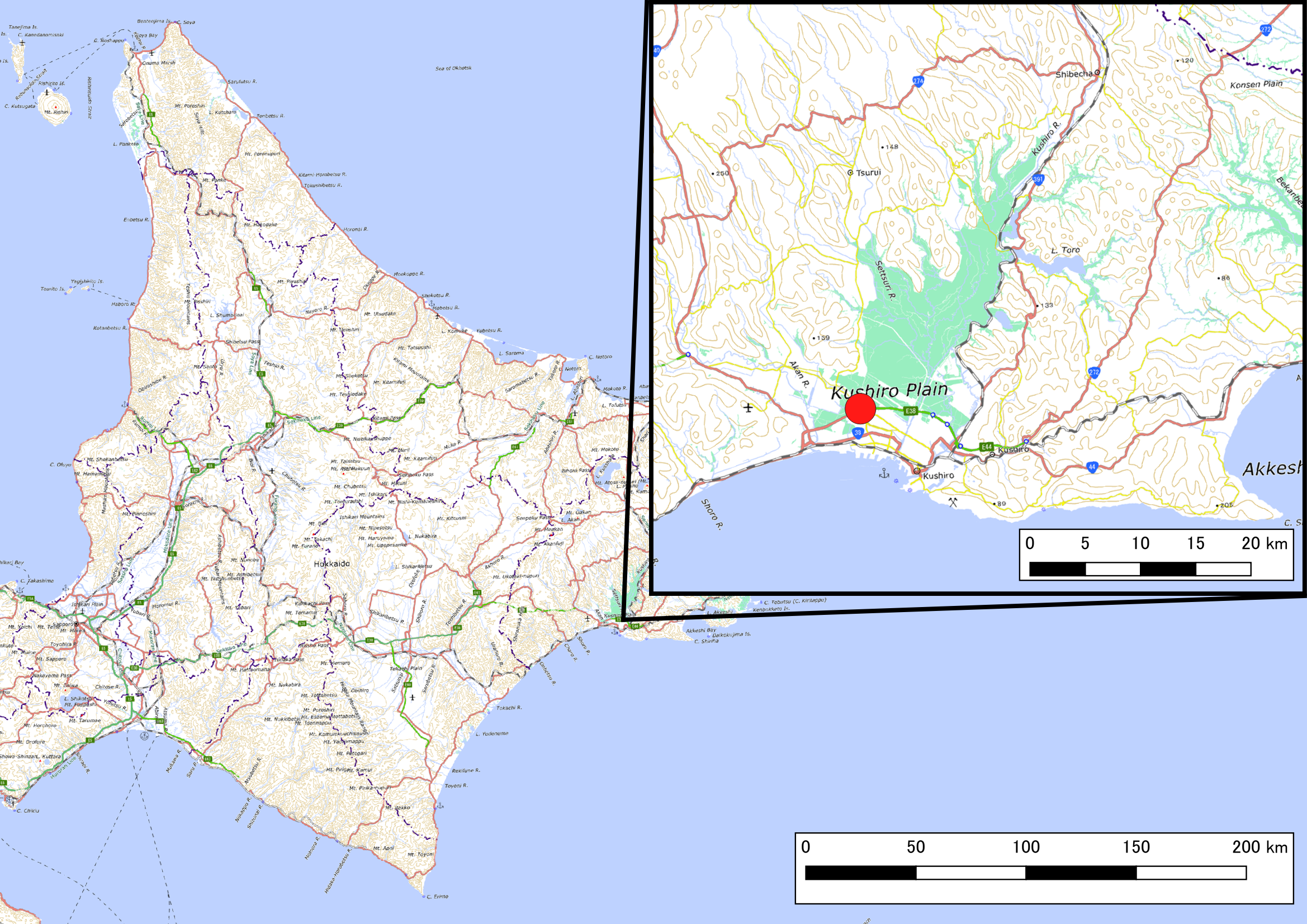

Supplement: Supplemental Information 1 — The red circle indicates the study area. This figure is based on the English map of Geospatial Information Authority of Japan (https://maps.gsi.go.jp/development/ichiran.html#english). [file peerj-08-9764-s001.png]

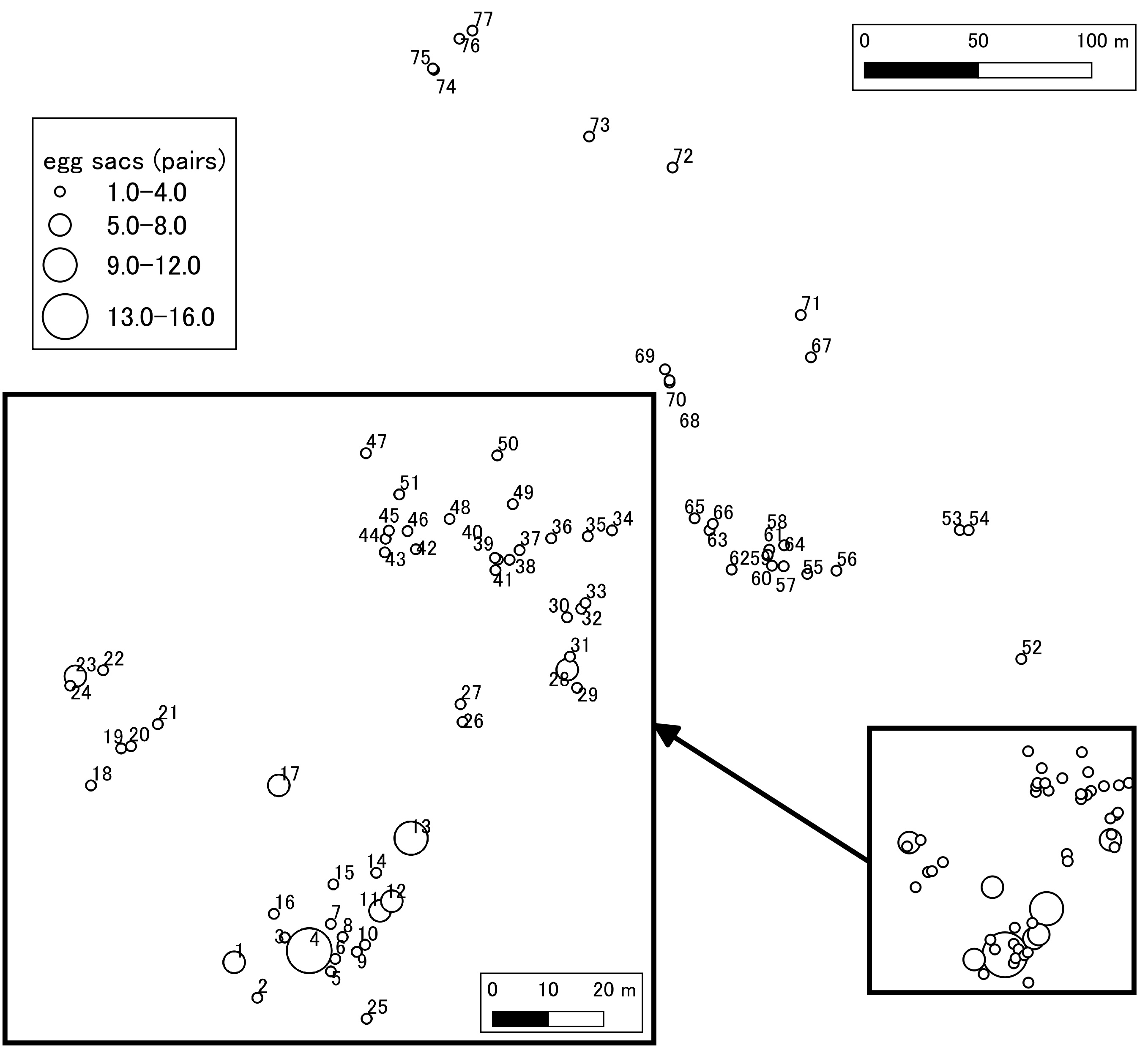

Supplement: Supplemental Information 2 — The size of the circle is changed according to the number of egg sacs found at each point. The identification number (1–77) corresponds to the “Site” of Supplemental Table S3, and by comparing it, the number of egg sacs and the time of discovery can be seen. [file peerj-08-9764-s002.png]
